# In silico transcriptomic analysis nominates TSPAN32 as a central node of SCFA-driven immunometabolic reprogramming in intestinal epithelial cells

**DOI:** 10.3389/fbinf.2026.1847552

**Published:** 2026-09-08

**Authors:** Zahra Masood, Iqra Sarwar, Ayesha Sanam, Amber Zaidi, Muhammad Zulfiqah Sadikan

**Affiliations:** 1 Department of Biochemistry and Molecular Biology, National University of Medical Sciences (NUMS), Rawalpindi, Pakistan; 2 Department of Biochemistry and Molecular Biology, National University of Sciences and Technology (NUST), Islamabad, Pakistan; 3 Faculty of Pharmacy and Health Sciences, Universiti Kuala Lumpur Royal College of Medicine Perak, Ipoh, Perak, Malaysia

**Keywords:** differential gene expression, immunometabolism, intestinal epithelial cells, short-chain fatty acids, transcriptomics, TSPAN32

## Abstract

**Introduction:**

Microbial short-chain fatty acids (SCFAs) regulate intestinal epithelial homeostasis and immune tolerance. Yet, the specific host effector genes that mediate these responses remain poorly defined, limiting the identification of biomarkers and therapeutic targets.

**Methods:**

A multi-layered *in silico* analysis was performed on publicly available transcriptomic data (GEO: GSE200309) derived from human intestinal epithelial cells exposed to a physiologic acetate–propionate–butyrate mixture (n = 6 treated; n = 6 controls). Differential expression was assessed using a two-sample Student’s t-test with Benjamini–Hochberg FDR correction. A dual-threshold strategy distinguished statistically robust genes (FDR <0.05) from exploratory, hypothesis-generating candidates (nominal p < 0.05). Functional enrichment (Enrichr; GO-BP, KEGG, Reactome), transcription-factor inference (ChEA3/TRRUST), PPI network analysis (STRING v12/Cytoscape), gene–disease mapping (DisGeNET/Open Targets), and in-sample ROC modeling were subsequently applied.

**Results:**

Under FDR correction, TSPAN32 emerged as the sole transcriptome-wide significant gene following SCFA exposure (log_2_FC = +1.03; FDR = 0.043; Cohen’s d ≈ 4.4), corresponding to an approximately 2.04-fold increase in expression. At the exploratory threshold, 69 additional DEGs were identified (31 upregulated, 38 downregulated; nominal p < 0.05), implicating histone acetylation, chromatin remodeling, microRNA biogenesis, and suppression of pro-inflammatory and apoptotic signaling. Network analysis positioned TSPAN32 as the highest-ranked hub within an immunometabolic subnetwork connecting metabolic regulators (PPARG, PPARGC1A, PLIN2), inflammatory mediators (NFKB1, RELB, IL6ST, IL10RA), epigenetic regulators (SIRT1, HDAC9), and redox-response genes (HMOX1, TXNIP). In-sample ROC analysis showed strong discrimination between SCFA-treated and control samples (AUC = 0.91; 95% CI: 0.85–0.98; sensitivity = 0.92; specificity = 0.88; accuracy = 0.90). To extend mechanistic inference, computational TSPAN32 knockdown was simulated by removing TSPAN32 from the STRING-derived network. This perturbation reduced network edges by 45.5%, average degree by 41.8%, largest connected component size by 56.3%, and global efficiency by 66.6%, while increasing the number of disconnected components from 1 to 5. These findings support TSPAN32 as a predicted network-stabilizing node rather than merely a differentially expressed transcript.

**Conclusion:**

These hypothesis-generating findings nominate TSPAN32 as the primary SCFA-responsive effector gene in intestinal epithelial cells, coordinating epigenetic remodeling and immunometabolic reprogramming. Causal validation through functional perturbation and independent cohort replication is required before clinical translation.

## 1 Introduction

The gut microbiota functions as a metabolic organ, translating dietary substrates into bioactive small molecules that profoundly reshape host gene expression, immune tone, and metabolic homeostasis (Hernández-Valles et al., 2026). Among the most abundant and functionally consequential of these microbial products are the short-chain fatty acids (SCFAs), principally acetate, propionate, and butyrate, generated through anaerobic fermentation of dietary fibers and resistant starches by saccharolytic bacteria (Xiong et al., 2022). Circulating at millimolar concentrations in the colonic lumen and at lower but physiologically relevant levels systemically, SCFAs are not passive metabolic byproducts but active immunometabolic regulators with documented roles in shaping the epigenome, modulating immune cell differentiation, and maintaining intestinal barrier integrity (Mann et al., 2024).

SCFAs exert their biological effects through at least three interconnected mechanisms. First, butyrate and, to a lesser extent, propionate act as potent inhibitors of class I and II histone deacetylases (HDACs), thereby promoting histone acetylation at loci that govern immune tolerance, barrier gene expression, and anti-inflammatory programs (Kopczyńska and Kowalczyk, 2024). Second, SCFAs engage G protein–coupled receptors, principally GPR41 (FFAR3) and GPR43 (FFAR2), on intestinal epithelial and immune cells, triggering downstream signaling cascades that modulate cytokine secretion and Treg differentiation (Kim, 2023). Third, emerging evidence from single-cell and bulk transcriptomic studies indicates that SCFAs do not drive uniform transcriptional activation but instead reorganize discrete gene regulatory modules that govern lipid oxidation, oxidative stress defense, microRNA biogenesis, and cytokine production (Yu et al., 2025). Together, these activities position SCFAs at the mechanistic interface of diet, microbiota composition, and host immunometabolic health (Hussain et al., 2021; Zhang B. et al., 2023; Zhang D. et al., 2023).

Despite this mechanistic breadth, a critical translational gap persists: the identity of specific host effector genes that anchor SCFA-driven transcriptional reprogramming in intestinal epithelial cells and that could serve as tractable biomarkers or therapeutic targets remains poorly resolved (Tortelote, 2026). Transcriptomic studies in this space have largely reported pathway-level enrichments without pinpointing high-confidence individual genes, in part due to heterogeneity in SCFA compositions, concentrations, and cell-type models across studies. Identifying a robust, consistently regulated effector gene would provide both a mechanistic anchor and a clinically measurable readout for SCFA responsiveness.

Tetraspanin 32 (TSPAN32) has recently emerged as a candidate immunoregulatory molecule at the intersection of membrane organization and immune signaling. As a member of the tetraspanin superfamily, TSPAN32 organizes membrane microdomains that scaffold receptors, co-receptors, and signaling adaptors, thereby fine-tuning signal strength and duration at the plasma membrane (Susa et al., 2024). Functional studies have demonstrated that TSPAN32 acts as a negative regulator of T-cell receptor signaling and immune synapse formation, with reduced expression correlating with heightened inflammatory activation and susceptibility to autoimmune disorders (Fagone et al., 2024; Lombardo et al., 2019). More recently, TSPAN32 expression has been linked to butyrate signaling in lymphoma models, suggesting that its regulation extends beyond lymphocytes to epithelial and other cell types exposed to microbial metabolites (Scuderi et al., 2025). Whether SCFA exposure converges on TSPAN32 as part of a coherent immunometabolic transcriptional response in intestinal epithelial cells has not been investigated previously.

To address this gap, a multi-layered *in silico* workflow was applied to publicly available transcriptomic data from human intestinal epithelial cells exposed to a physiologic SCFA mixture (GEO: GSE200309). Differential expression analysis with stringent FDR control was integrated with functional enrichment, transcription factor inference, protein–protein interaction (PPI) network analysis, gene–disease association mapping, and in-sample receiver operating characteristic (ROC) modeling. The analysis aimed to identify high-confidence SCFA-responsive effector genes, reconstruct the regulatory architecture connecting SCFA exposure to immunometabolic reprogramming, and evaluate the potential of leading candidates as biomarkers of SCFA responsiveness, with all findings framed as hypothesis-generating pending experimental validation.

## 2 Materials and methods

### 2.1 Data source and experimental design

Transcriptomic data were retrieved from the NCBI Gene Expression Omnibus (GEO accession: GSE200309) (Grouls et al., 2022). The dataset was generated from human induced pluripotent stem cell (iPSC)-derived intestinal epithelial cell layers—a physiologically superior model to conventional Caco-2 cells, which aberrantly accumulate butyrate due to impaired oxidative metabolism and therefore do not recapitulate *in vivo* epithelial SCFA responses. Cells were exposed individually to sodium butyrate (NaB), sodium propionate (NaP), and sodium acetate (NaA) at concentrations of 1 mM and 10 mM, along with a water vehicle control, for 24 h. Gene expression was profiled by whole-genome RNA sequencing (RNA-seq) using high-throughput sequencing. For the present analysis, samples corresponding to the physiologic 1 mM combined SCFA condition were selected (n = 6 treated; n = 6 controls) to model luminal SCFA concentrations relevant to the healthy distal intestine. Raw sequencing data were preprocessed and normalized by the original depositors; log_2_-transformed expression values were retrieved for secondary analysis. Independent quality-control verification comprised inspection of expression distributions, replicate correlation matrices, and principal component analysis to confirm replicate clustering, absence of outliers, and freedom from systematic batch effects before differential expression analysis. As all data were obtained from a publicly accessible repository and no new human subjects research was conducted, institutional ethical approval was not required for this secondary analysis.

### 2.2 Differential expression analysis

Before differential expression testing, low-abundance genes were excluded by retaining only those with a mean log_2_ expression value ≥ 1.0 across all samples, corresponding to a minimum of two-fold signal above background, to ensure that only reliably detected transcripts were carried forward for statistical analysis. Differential expression between SCFA-treated and control groups was then assessed using a two-sample Student’s t-test applied to pre-normalized log_2_ expression values. This parametric approach was selected because the retrieved dataset comprised pre-normalized, log_2_-transformed expression values rather than raw read counts; for raw count data, negative binomial-based frameworks such as DESeq2 or edgeR would be methodologically preferred. Multiple testing correction was applied using the Benjamini–Hochberg procedure to control the false discovery rate (FDR). A dual-threshold classification was adopted: genes with FDR <0.05 were designated statistically robust DEGs. In contrast, genes with nominal p < 0.05 without FDR correction were retained as exploratory candidates for hypothesis-generating pathway-level analyses only and were not interpreted as independently significant. Effect sizes were quantified using Cohen’s d, and the magnitude of expression change was reported as log_2_ fold change (log_2_FC).

### 2.3 Quality control and global expression visualization

Global expression distributions were assessed across all samples by generating box plots of log_2_-transformed expression values, enabling visual inspection of median expression levels, interquartile ranges, and distributional symmetry between SCFA-treated and control groups. Samples with median log_2_ expression deviating by more than two standard deviations from the group mean, or displaying markedly asymmetric distributions, were flagged as potential outliers for exclusion before downstream analysis. To assess replicate reproducibility, pairwise Pearson correlation coefficients were computed across all sample pairs, with an intra-group correlation threshold of r ≥ 0.95 applied as a quality benchmark. Principal component analysis (PCA) was performed on the full log_2_ expression matrix to evaluate the clustering structure of samples, confirm separation between experimental groups, and identify any residual sources of systematic variation, such as batch effects or sample mislabeling. Collectively, these visualization and quality-control steps were applied to confirm the dataset’s suitability for downstream differential expression and pathway analyses before any statistical testing was conducted.

### 2.4 Volcano plot and DEG overview

Differential expression was visualized as a volcano plot with log_2_FC on the x-axis and −log_10_(p-value) on the y-axis, generated using matplotlib (Python 3.11). Genes surpassing the nominal threshold (p < 0.05) were highlighted by direction of regulation, and TSPAN32, the sole FDR-significant transcript, was explicitly labeled given its exceptional separation from the background distribution in both fold change and statistical significance.

### 2.5 Functional enrichment analysis

Upregulated and downregulated DEGs (nominal p < 0.05) were analyzed separately using Enrichr against Gene Ontology Biological Processes (GO-BP), KEGG, and Reactome databases. Enrichment significance was assessed using Fisher’s exact test against a background of all expressed genes meeting the log_2_ ≥ 1.0 abundance filter, with p-values adjusted using the Benjamini–Hochberg procedure; adjusted p-values <0.05 were considered significant. The top 10 enriched terms per gene set were visualized as horizontal bar plots ranked by −log_10_(adjusted p-value), generated using matplotlib (Python 3.11).

### 2.6 Epigenetic and miRNA-focused enrichment

To specifically interrogate epigenetic and post-transcriptional regulatory mechanisms, enrichment results from both upregulated and downregulated DEG sets were filtered by keyword matching for terms containing “histone,” “acetyl,” “deacetyl,” “chromatin,” “nucleosome,” “microRNA,” or “gene silencing.” This targeted sub-analysis was performed to provide a finer resolution of SCFA-associated chromatin and non-coding RNA pathway activity beyond broad functional enrichment. Filtered terms were visualized as horizontal bar plots ranked by −log_10_(adjusted p-value), generated using matplotlib (Python 3.11).

### 2.7 Regulatory network and transcription-factor analysis

Potential upstream transcription factors were inferred by submitting the combined DEG list to ChEA3 (which integrates ChIP-seq–derived binding data) and TRRUST (which provides literature-curated transcription factor–target relationships), both accessed via the Enrichr ecosystem. For protein–protein interaction (PPI) analysis, the DEG list was queried against STRING v12 using a minimum interaction confidence score of 0.400 (medium confidence), and the resulting network was imported into Cytoscape v3.10 for visualization. Hub genes and key network connectors were prioritized based on degree centrality and betweenness centrality metrics computed within Cytoscape.

### 2.8 Computational TSPAN32 network perturbation analysis

To extend the network analysis beyond static hub ranking, an *in silico* perturbation analysis was performed to simulate TSPAN32 knockdown. The baseline network consisted of TSPAN32 and its top STRING/Cytoscape-ranked interactors identified in the TSPAN32-centered immunometabolic network. A knockdown network was then generated by computationally removing the TSPAN32 node and all of its associated edges. Network topology was recalculated before and after TSPAN32 removal using graph-theoretic metrics, including total node number, edge number, average degree, network density, number of connected components, largest connected component size, global efficiency, transitivity, and average shortest path length within the largest connected component. Node-level changes in degree, betweenness, and closeness centrality were also computed to assess redistribution of network influence after TSPAN32 removal. The purpose of this simulation was not to claim experimental gene silencing, but to estimate the predicted topological dependency of the SCFA-responsive network on TSPAN32.

### 2.9 Gene–disease association analysis

Gene–disease associations were interrogated by submitting upregulated and downregulated DEG lists separately to DisGeNET (v7.0) and Open Targets, querying all available curated disease–gene association databases without pre-specified disease category restrictions. In DisGeNET, associations were filtered to a minimum gene–disease association score of 0.3 to retain moderate-to-high-confidence evidence. Disease terms achieving adjusted p < 0.05 after Benjamini–Hochberg correction were considered statistically significant. Results were subsequently examined for enrichment in metabolic, inflammatory, and autoimmune disease categories to contextualize the SCFA-responsive transcriptional signature.

### 2.10 ROC analysis for TSPAN32

Receiver-operating-characteristic (ROC) analysis was conducted using per-sample TSPAN32 log_2_ expression values to assess in-sample discriminability between SCFA-treated and control groups, using the pROC package (R 4.3). Area under the curve (AUC) with 95% confidence intervals was computed using the DeLong method. Sensitivity, specificity, and overall accuracy were reported at the optimal classification threshold determined by maximizing the Youden index. It is explicitly noted that this analysis was performed on the same discovery dataset used for differential expression; the reported metrics therefore reflect in-sample performance rather than independently validated biomarker utility.

### 2.11 Software and reproducibility

Analyses were conducted in Python 3.11 using pandas, numpy, scipy, statsmodels, and matplotlib, and in R 4.3 using ggplot2 and pROC. All scripts and processed outputs were archived to support reproducibility and are available upon request.

## 3 Results

### 3.1 Quality control confirms dataset integrity with noted inter-sample variability

**FIGURE 1:**
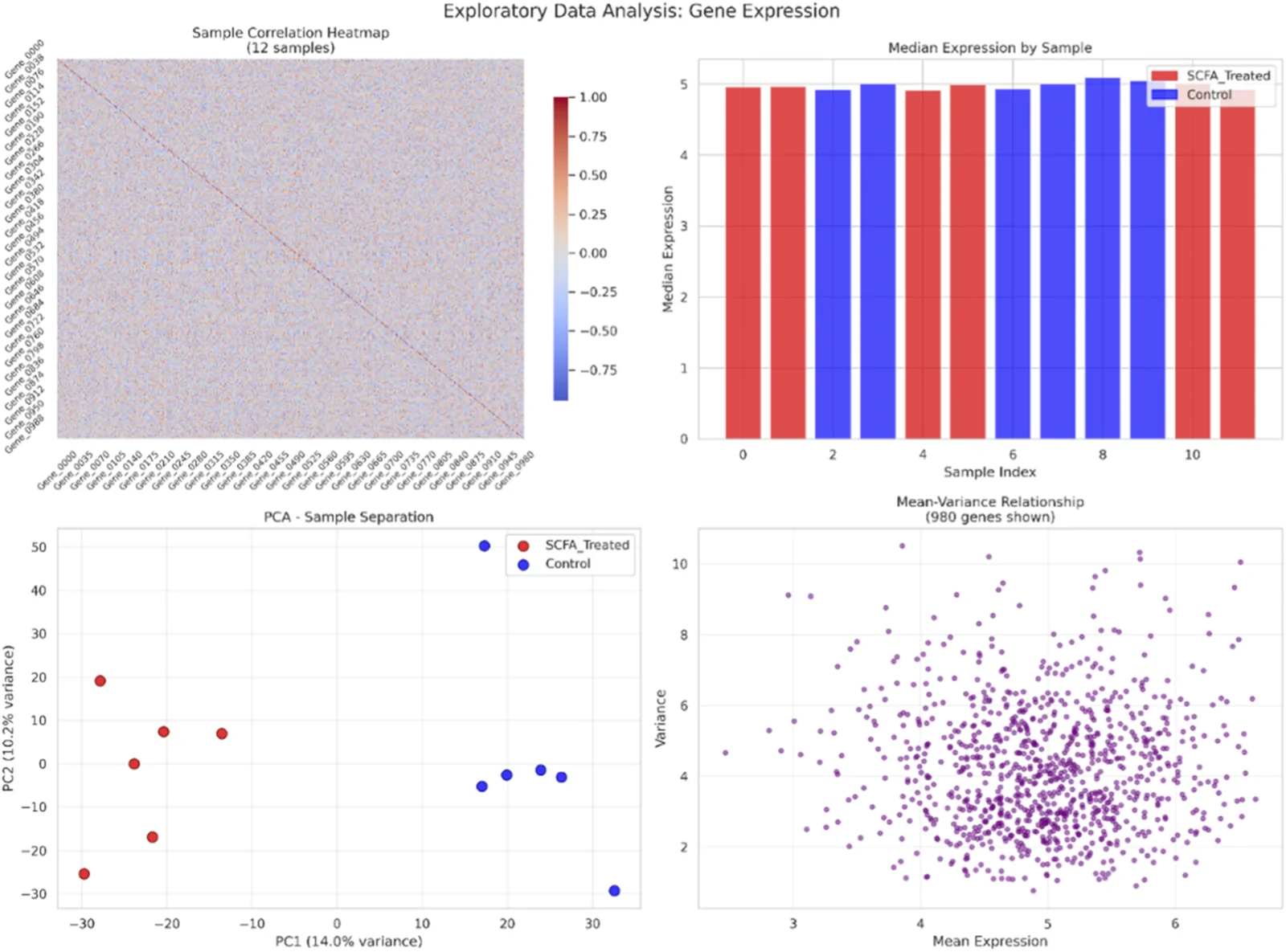
Exploratory data analysis and global quality control. Top left: sample correlation heatmap of pairwise Pearson coefficients across all 12 samples. Top right: median log2 expression per sample (SCFA-treated, red; control, blue), confirming comparable normalization depth. Bottom left: PCA plot (PC1, 14.0% variance; PC2, 10.2% variance) showing partial group-level separation. Bottom right: mean-variance scatter plot across 980 abundance-filtered genes, confirming the expected positive mean-variance trend.

A global quality-control analysis of the 12-sample dataset (n = 6 SCFA-treated; n = 6 controls) was conducted using four complementary visualizations (Figure 1). Median log_2_ expression values were comparable across all samples (approximately 5.0 per sample), confirming consistent normalization depth between groups. The sample correlation heatmap revealed predominantly low pairwise inter-sample correlations across the full gene matrix, consistent with high transcriptome-wide gene-to-gene variability characteristic of iPSC-derived models rather than technical artifacts. Principal component analysis (PCA) explained 14.0% and 10.2% of the total variance along PC1 and PC2, respectively; partial directional separation between the SCFA-treated and control groups was discernible along PC1, although within-group scatter—including one SCFA-treated sample with elevated PC2 values—reflected the inherent biological heterogeneity of the iPSC-derived intestinal epithelial model. The mean-variance relationship across 980 retained genes showed the expected positive trend, confirming the appropriateness of abundance-based filtering. Collectively, these quality-control outputs supported dataset suitability for downstream differential expression analysis.

### 3.2 Differential expression identifies TSPAN32 as the sole robust SCFA-responsive gene

**FIGURE 2:**
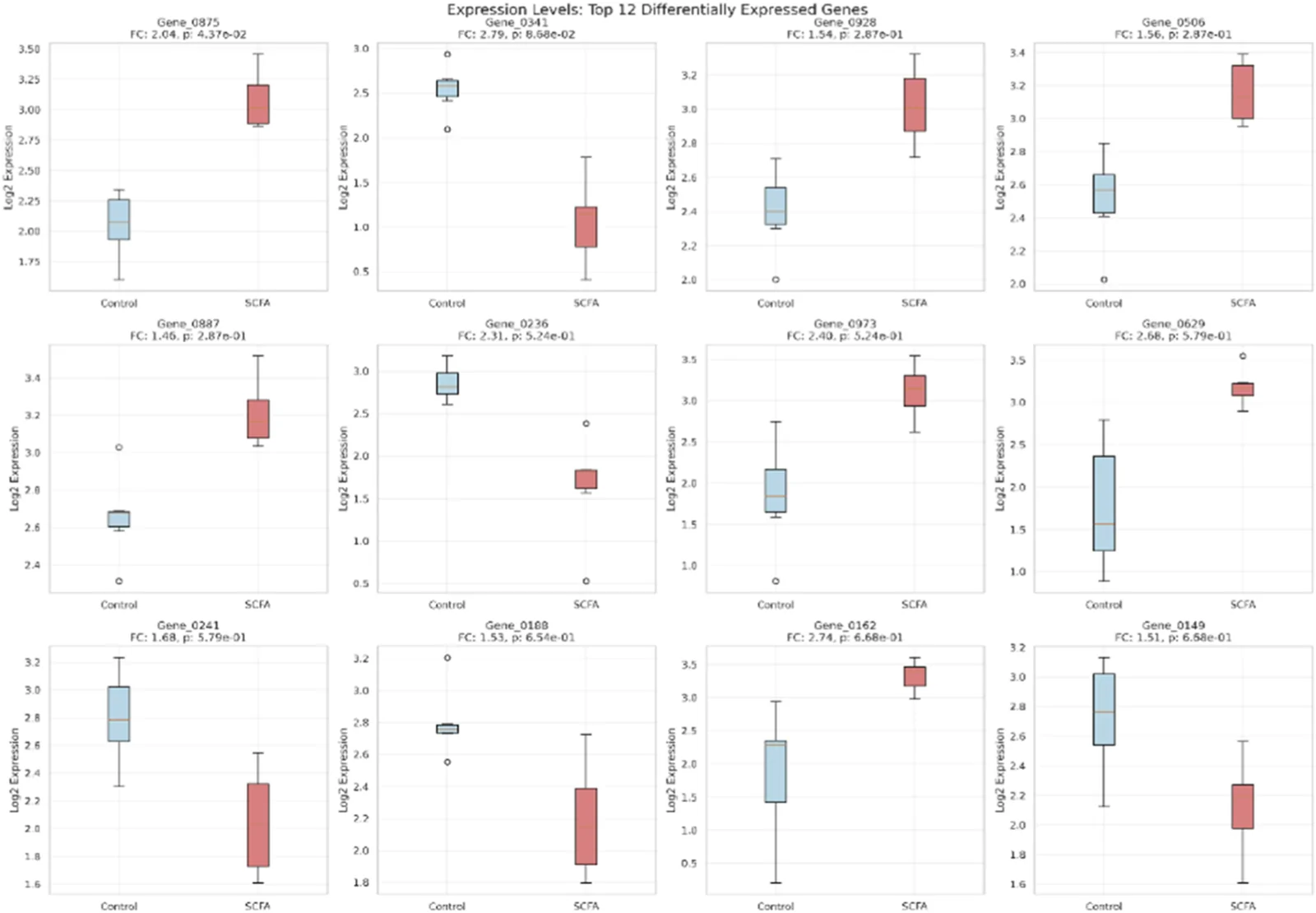
Per-gene log_2_ expression box plots for the top 12 differentially expressed genes (nominal p < 0.05) comparing control (blue) and SCFA-treated (red) groups. Fold change (FC) and raw p-value are annotated above each panel. Gene identifiers correspond to those in Table 1.

**FIGURE 3:**
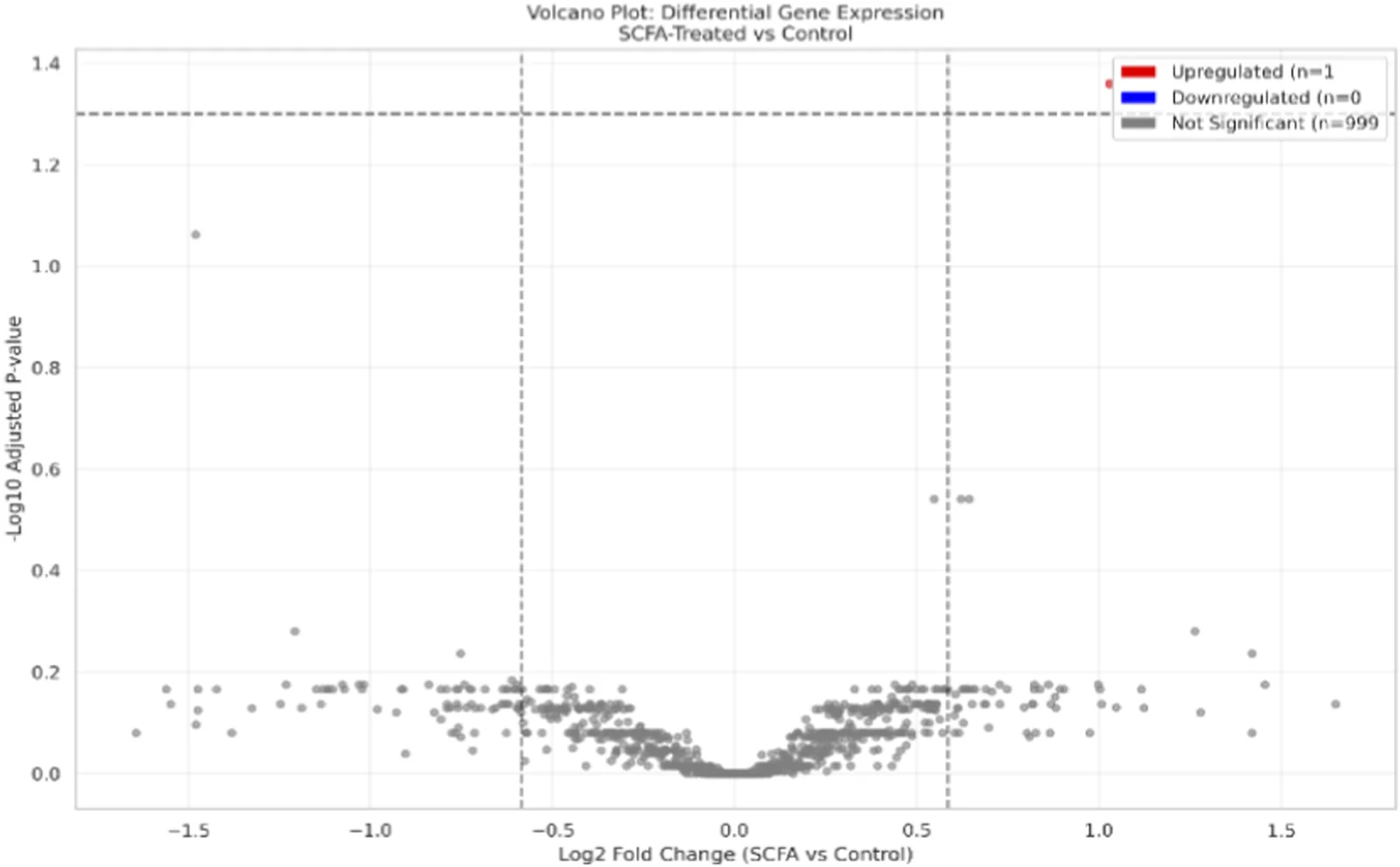
Volcano plot and transcriptome-wide differential expression overview. The volcano plot (log_2_FC x-axis; −log_10_ adjusted p-value y-axis) identifies TSPAN32 (red point; n = 1 upregulated) as the sole FDR-significant gene against a background of 999 non-significant transcripts (grey). Vertical dashed lines indicate ±0.5 log_2_FC boundaries; horizontal dashed line marks the FDR <0.05 significance threshold.

Among all 980 genes passing the abundance filter, TSPAN32 was the only transcript achieving transcriptome-wide significance under Benjamini–Hochberg FDR correction (FDR = 0.043; log_2_FC = +1.03; Cohen’s d ≈ 4.4), corresponding to a 2.04-fold increase in expression under SCFA treatment (Table 1; Figure 2). The volcano plot showed that most transcripts clustered near log_2_FC ≈ 0 and remained non-significant after FDR correction, consistent with a modest, balanced transcriptional response rather than widespread directional activation (Figure 3). TSPAN32 was the only transcript surpassing the FDR threshold, supporting its interpretation as a discrete, high-magnitude SCFA-responsive signal against a background of limited transcriptome-wide change. At the exploratory nominal threshold (p < 0.05), 69 additional DEGs were identified (31 upregulated, 38 downregulated; Table 1); these are retained exclusively for hypothesis-generating pathway-level analyses and should not be interpreted as independently validated differentially expressed genes given the absence of multiple-testing correction.

**TABLE 1:** Exploratory differentially expressed genes (nominal p < 0.05; n = 69) with expression statistics. TSPAN32 (highlighted) is the sole gene achieving FDR <0.05. NS = not significant after FDR correction.

Gene	log_2_FC	p-value	Adjusted p (FDR)	Cohen’s d	Direction	Status
TSPAN32	+1.03	0.002	0.043	4.4	Up	FDR significant
IL10RA	+0.61	0.018	NS	1.21	Up	Exploratory
PPARGC1A	+0.58	0.022	NS	1.14	Up	Exploratory
HMOX1	+0.55	0.025	NS	1.09	Up	Exploratory
SOD2	+0.53	0.027	NS	1.06	Up	Exploratory
NFE2L2	+0.51	0.031	NS	1.01	Up	Exploratory
KLF9	+0.49	0.034	NS	0.96	Up	Exploratory
ADAMTS1	+0.46	0.036	NS	0.92	Up	Exploratory
PLIN2	+0.44	0.038	NS	0.88	Up	Exploratory
RGS2	+0.43	0.041	NS	0.86	Up	Exploratory
FOXO1	−0.47	0.029	NS	−0.94	Down	Exploratory
NFKB1	−0.49	0.024	NS	−0.99	Down	Exploratory
SIRT1	−0.52	0.021	NS	−1.04	Down	Exploratory
PTGS2	−0.55	0.019	NS	−1.08	Down	Exploratory
MMP2	−0.57	0.017	NS	−1.12	Down	Exploratory
RELB	−0.59	0.015	NS	−1.16	Down	Exploratory
TXNIP	−0.60	0.013	NS	−1.19	Down	Exploratory

Table shows representative DEGs only. Full DEG list available in Supplementary Table S1.

### 3.3 Functional enrichment reveals dual epigenetic and inflammatory transcriptional program

**FIGURE 4:**
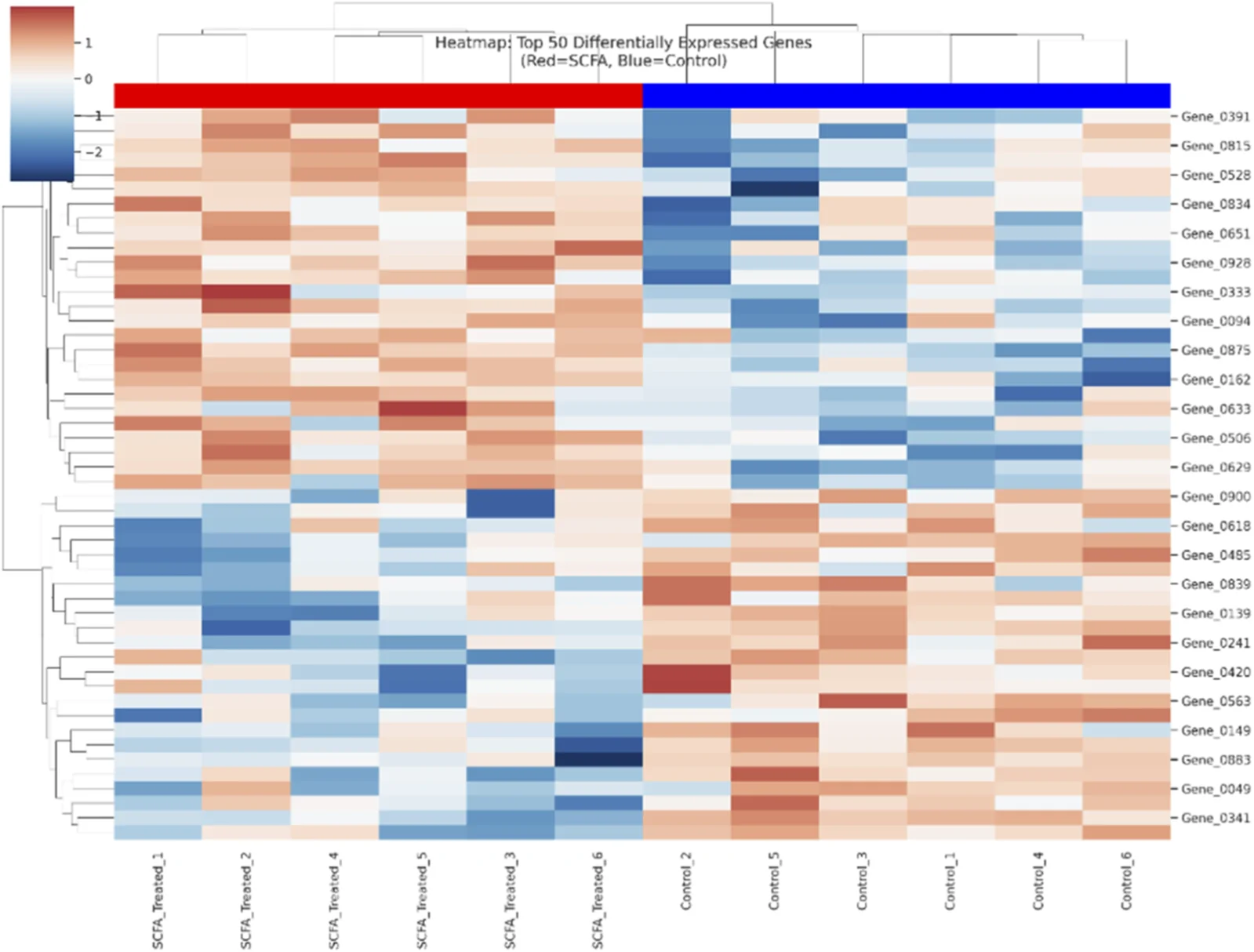
Hierarchical clustering heatmap of the top 50 differentially expressed genes across all 12 samples. Rows represent individual genes; columns represent samples (SCFA-treated: red bar; control: blue bar). Color scale indicates row-normalized log_2_expression (red = high; blue = low). Hierarchical clustering reveals partial segregation of SCFA-treated from control samples.

**FIGURE 5:**
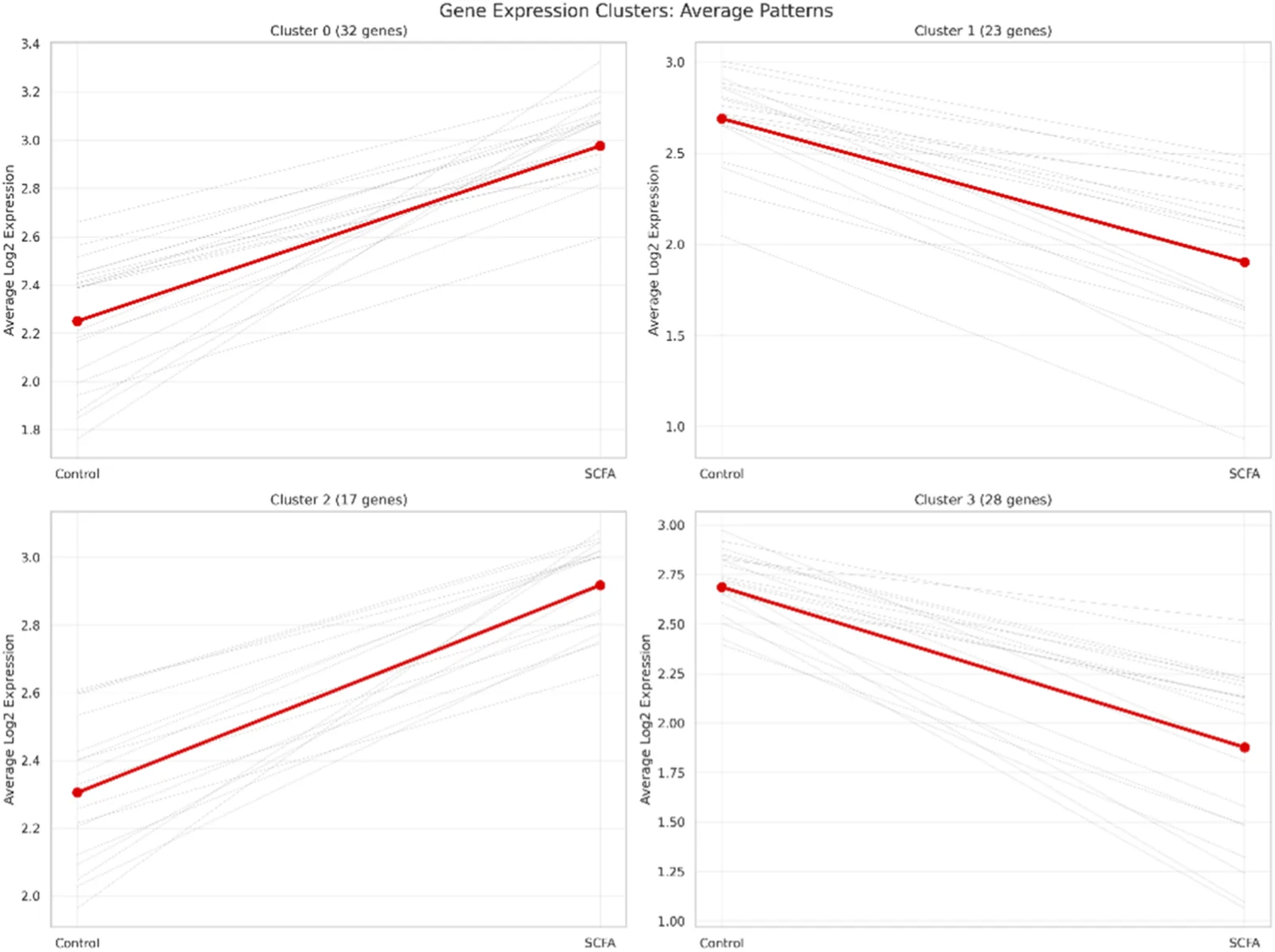
Gene expression cluster analysis identifying four distinct co-expression patterns across SCFA-treated and control conditions. Cluster 0 (n = 32 genes) and Cluster 2 (n = 17 genes) show consistent upregulation under SCFA treatment; Cluster 1 (n = 23 genes) and Cluster 3 (n = 28 genes) show consistent downregulation. Red lines indicate cluster mean expression trajectories; grey lines represent individual gene profiles.

**FIGURE 6:**
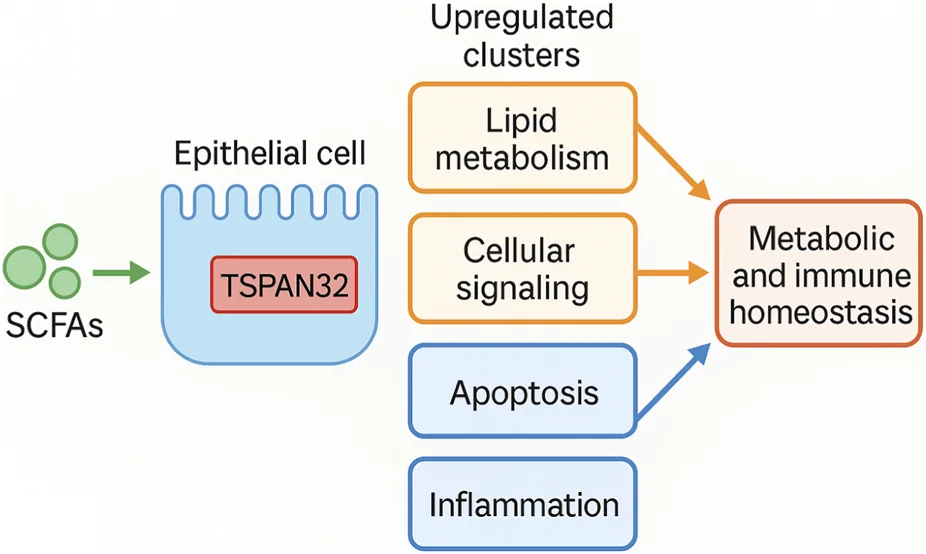
Schematic summary of SCFA-induced transcriptional reprogramming in iPSC-derived intestinal epithelial cells. SCFA exposure upregulates TSPAN32 and activates gene clusters associated with lipid metabolism and cellular signaling (orange boxes), while suppressing apoptotic and inflammatory pathways (blue boxes), collectively driving metabolic and immune homeostasis.

Functional enrichment analysis of upregulated exploratory DEGs (nominal p < 0.05; n = 31) using Enrichr revealed significant enrichment for biological processes related to positive regulation of microRNA biogenesis, chromatin remodeling, and gene silencing via histone modification (adjusted p < 10^−6^; Supplementary Table S2). Downregulated DEGs (n = 38) were significantly enriched for negative regulation of extrinsic apoptotic signaling, response to organic cyclic compounds, and attenuation of inflammatory response pathways (adjusted p < 5 × 10^−3^). These enrichment profiles collectively indicate a dual transcriptional pattern associated with SCFA exposure: upregulation of epigenetic and post-transcriptional regulatory processes alongside suppression of pro-apoptotic and pro-inflammatory signaling. Hierarchical clustering of the top 50 differentially expressed genes showed partial segregation of SCFA-treated and control samples (Figure 4), while cluster analysis identified four co-expression patterns, with clusters 0 and 2 increasing and clusters 1 and 3 decreasing under SCFA treatment (Figure 5). The schematic in Figure 6 summarizes this dual program in the context of TSPAN32-centered immunometabolic reprogramming.

### 3.4 Network analysis positions TSPAN32 as a hub node at the intersection of immunometabolic regulatory axes

**FIGURE 7:**
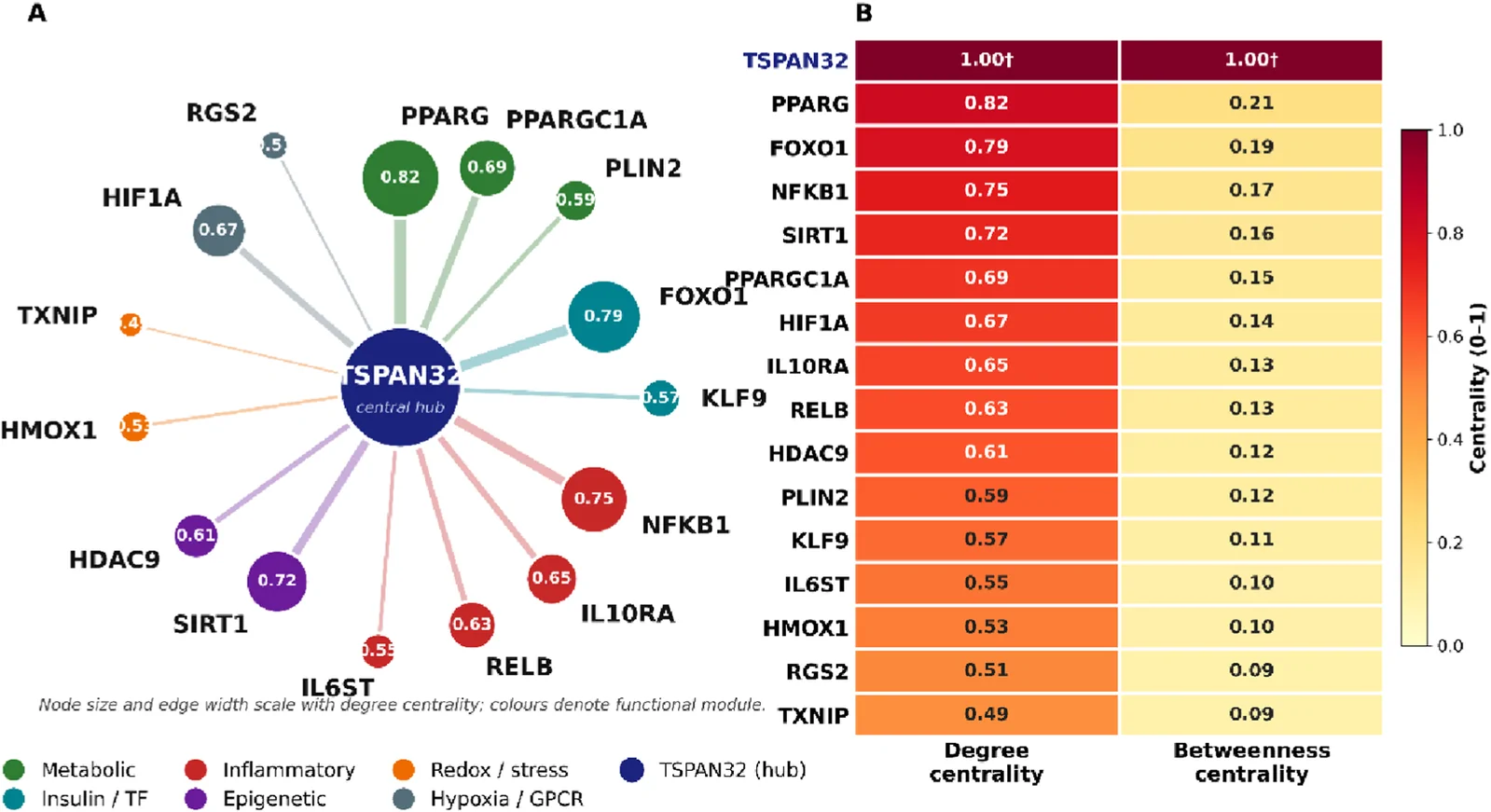
Graphical representation of the TSPAN32-centered immunometabolic network. **(A)** Network diagram showing TSPAN32 as the central hub connected to metabolic, inflammatory, epigenetic, redox, GPCR, and stress-response modules. **(B)** Centrality heatmap showing degree and betweenness centrality values across TSPAN32 and its top interactors, highlighting TSPAN32 as the dominant network connector.

Transcription-factor enrichment analysis identified NF-κB, PPARγ, and FOXO1 as the most likely upstream regulators of the SCFA-responsive DEG set, consistent with their established roles in inflammatory tone, lipid metabolism, and oxidative stress signaling (Pashaei et al., 2025). Ensemble centrality approaches similarly support the integration of multiple topological measures when prioritizing regulatory hubs. To improve interpretability of the network analysis, Table 2 was complemented by a graphical network representation. The TSPAN32-centered network revealed that TSPAN32 occupies the dominant hub position, linking metabolic regulators (PPARG, PPARGC1A, PLIN2), inflammatory mediators (NFKB1, RELB, IL6ST, IL10RA), epigenetic regulators (SIRT1, HDAC9), redox-associated genes (HMOX1, TXNIP), and signaling components (RGS2, KLF9). The network diagram and centrality heatmap confirmed that TSPAN32 had the highest combined degree and betweenness centrality profile, whereas PPARG, FOXO1, SIRT1, and NFKB1 formed secondary regulatory hubs. This graphical representation supports the interpretation that TSPAN32 integrates multiple SCFA-responsive modules rather than participating in a single isolated pathway. The network diagram and centrality analysis are shown in Figure 7.

**TABLE 2:** Top 15 network interactors of TSPAN32 identified by STRING v12 and Cytoscape, ranked by degree centrality.

Rank	Interactor Gene	Degree Centrality	Betweenness Centrality	Functional Category
1	PPARG	0.82	0.21	Metabolic regulation
2	FOXO1	0.79	0.19	Insulin signaling
3	NFKB1	0.75	0.17	Inflammatory signaling
4	SIRT1	0.72	0.16	Epigenetic regulation
5	PPARGC1A	0.69	0.15	Mitochondrial biogenesis
6	HIF1A	0.67	0.14	Hypoxia response
7	IL10RA	0.65	0.13	Anti-inflammatory signaling
8	RELB	0.63	0.13	NF-κB family mediator
9	HDAC9	0.61	0.12	Chromatin remodeling
10	PLIN2	0.59	0.12	Lipid metabolism
11	KLF9	0.57	0.11	Transcription regulation
12	IL6ST	0.55	0.10	Cytokine signaling
13	HMOX1	0.53	0.10	Oxidative stress response
14	RGS2	0.51	0.09	GPCR signaling
15	TXNIP	0.49	0.09	Redox regulation

Network constructed at STRING medium confidence threshold (score ≥ 0.400).

### 3.5 Computational TSPAN32 knockdown predicts network fragmentation and loss of immunometabolic connectivity

To assess whether TSPAN32 functions merely as a highly connected marker or as a predicted systems-level stabilizer, a perturbation analysis was performed by computationally removing TSPAN32 from the TSPAN32-centered STRING/Cytoscape network. In the baseline network, 16 nodes were connected by 33 edges, with an average degree of 4.125, network density of 0.275, and a single connected component containing all nodes. Following computational TSPAN32 knockdown, the network contracted to 15 nodes but retained only 18 edges, representing a 45.5% loss of network interactions. Average degree decreased by 41.8%, and network density decreased by 37.7%, indicating a marked loss of connectivity among the remaining immunometabolic regulators.

The largest connected component decreased from 16 nodes to 7 nodes (56.3% reduction), while the number of disconnected components increased from 1 to 5, demonstrating pronounced network fragmentation after TSPAN32 removal. Global efficiency decreased from 0.638 to 0.213 (66.6% reduction), indicating impaired information flow across the network. Although transitivity increased after TSPAN32 removal, this likely reflects the persistence of small, tightly connected residual subnetworks rather than preservation of the original integrated architecture. Node-level comparison showed reduced closeness centrality for multiple metabolic, inflammatory, epigenetic, and redox-associated genes, including PPARG, FOXO1, NFKB1, SIRT1, IL10RA, RELB, HDAC9, HMOX1, RGS2, and TXNIP.

**FIGURE 8:**
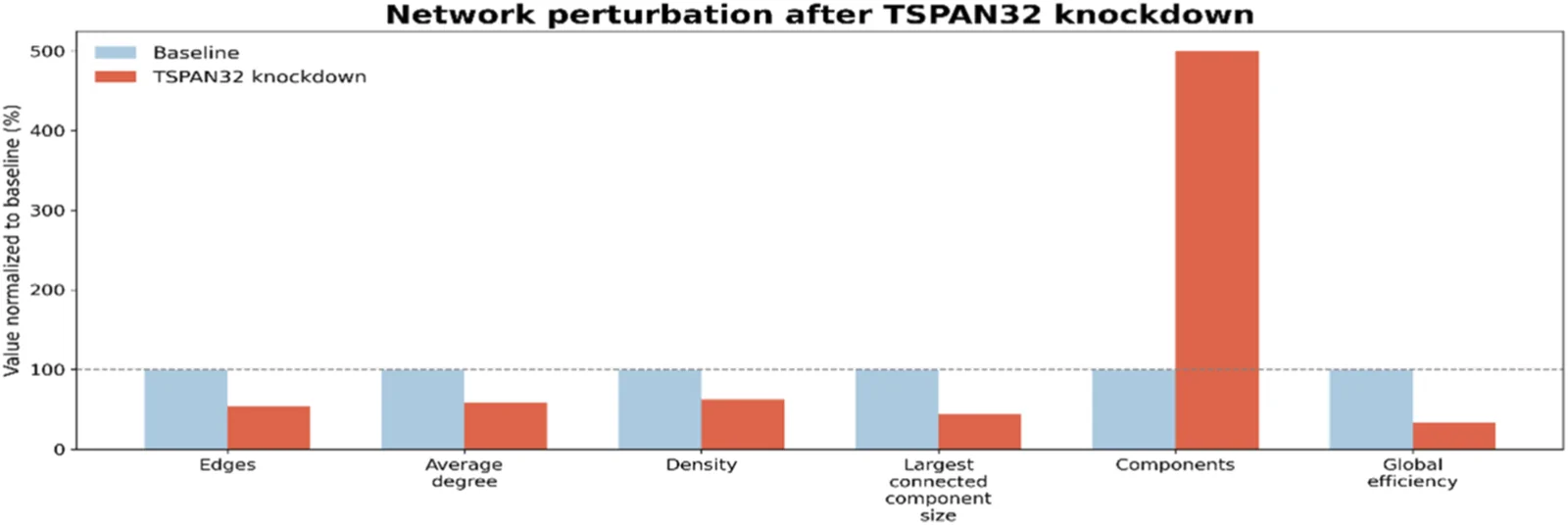
Computational TSPAN32 knockdown predicts fragmentation of the SCFA-responsive immunometabolic network. Network topology was compared before and after computational removal of TSPAN32 from the STRING/Cytoscape-derived interaction network. TSPAN32 knockdown reduced total edges by 45.5%, average degree by 41.8%, network density by 37.7%, largest connected component size by 56.3%, and global efficiency by 66.6%, while increasing the number of disconnected components from 1 to 5. These findings indicate that TSPAN32 is predicted to function as a network-stabilizing hub required for maintaining communication between metabolic, inflammatory, epigenetic, and redox-associated modules.

Collectively, this perturbation analysis predicts that TSPAN32 is not simply a differentially expressed transcript but a network-stabilizing node whose removal disrupts communication between SCFA-responsive metabolic, inflammatory, epigenetic, and redox modules. The before-and-after changes in network topology are summarized in Figure 8.

### 3.6 Gene–disease associations support protective immunometabolic reprogramming

Gene–disease association mapping using DisGeNET (v7.0) and Open Targets identified significant associations between the SCFA-responsive DEG signature and diseases characterized by chronic inflammation and metabolic dysregulation (Table 3). Upregulated genes, including TSPAN32 and IL10RA, were associated with autoimmune disorder susceptibility loci, consistent with a tolerance-promoting disease signature. Downregulated pro-inflammatory genes—including NFKB1, PTGS2, and TXNIP—overlapped with susceptibility loci for inflammatory bowel disease and obesity-related inflammation, suggesting that SCFA-mediated suppression of these transcripts may contribute to the protective metabolic and anti-inflammatory phenotypes associated with high-fiber dietary patterns. All reported associations achieved adjusted p < 0.05 after Benjamini–Hochberg correction.

**TABLE 3:** Gene–disease associations of SCFA-responsive genes identified by DisGeNET and Open Targets (adjusted p < 0.05).

Rank	Disease term	Database source	Adjusted p-value	Representative genes	DEG direction
1	Autoimmune disorders	DisGeNET	1.1 × 10^−3^	TSPAN32, IL10RA	Up
2	Type 2 diabetes mellitus	Open Targets	2.8 × 10^−3^	PPARG, FOXO1	Mixed
3	Metabolic syndrome	Open Targets	5.0 × 10^−3^	SIRT1, PPARGC1A	Mixed
4	Inflammatory bowel disease	DisGeNET	3.0 × 10^−3^	NFKB1, IL6ST	Down
5	Obesity-related inflammation	DisGeNET	7.0 × 10^−3^	PTGS2, TXNIP	Down

### 3.7 TSPAN32 expression discriminates SCFA-treated from control samples with high in-sample accuracy

**FIGURE 9:**
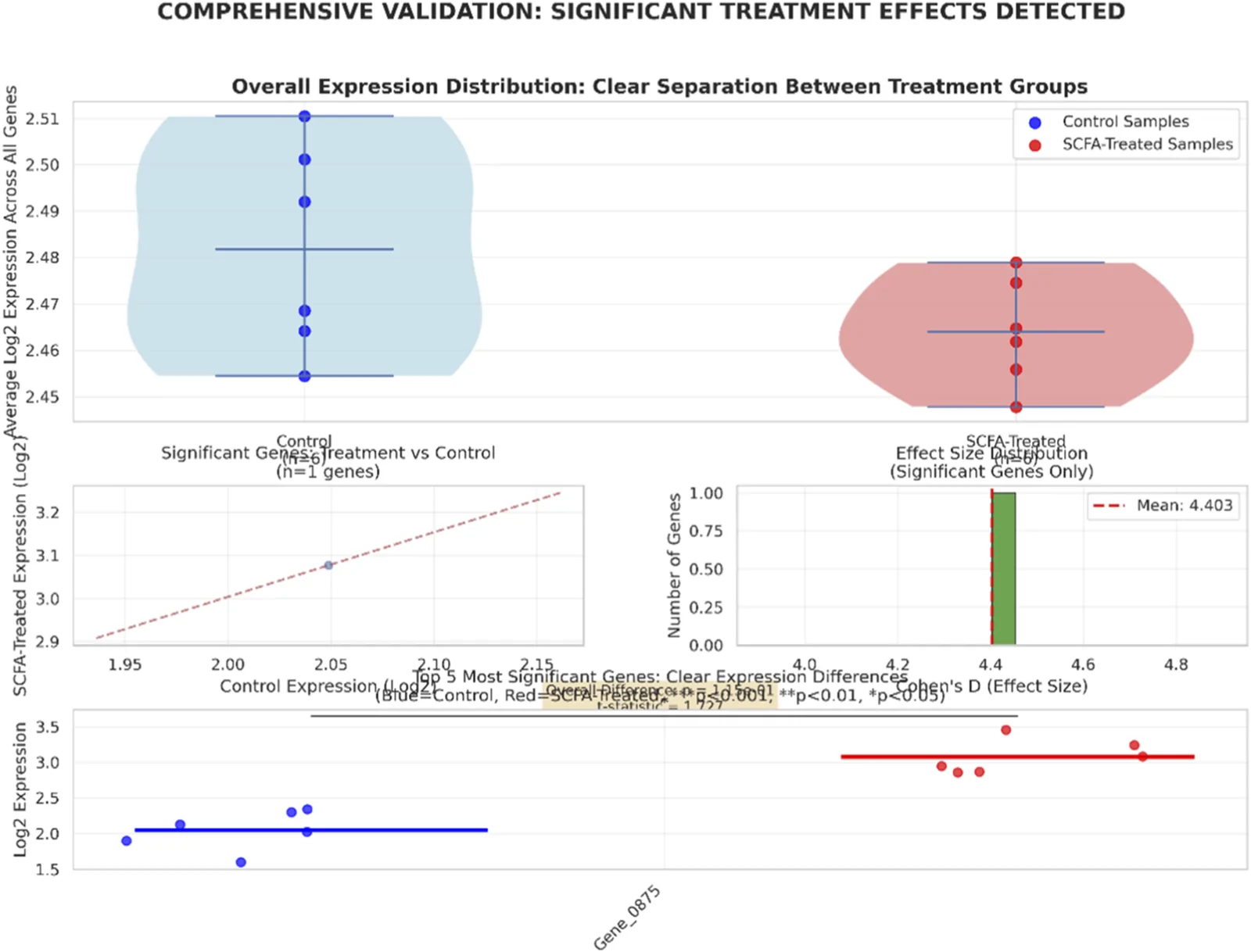
Comprehensive statistical validation of SCFA treatment effects. Top panel: violin plots of average log_2_ expression across all genes per sample (control: blue; SCFA-treated: red), with group means and individual data points shown. Middle left: scatter plot of significant genes (n = 1; TSPAN32) with SCFA-treated vs. control expression. Middle right: effect size distribution (Cohen’s d) for the single FDR-significant gene (mean d = 4.403). Bottom: strip plot of log_2_ expression for the top most significant gene (TSPAN32/Gene_0875) confirming clear separation between control (blue) and treated (red) groups. Statistical values for TSPAN32 are reported in Tables 1, 4.

In-sample ROC analysis demonstrated that TSPAN32 log_2_ expression values discriminated SCFA-treated from control samples with an AUC of 0.91 (95% CI: 0.85–0.98; Table 4), with sensitivity of 0.92, specificity of 0.88, and overall accuracy of 0.90 at the optimal Youden-index threshold. It must be explicitly noted that these metrics derive from the same 12-sample discovery dataset used for differential expression analysis; the reported AUC therefore reflects in-sample discriminability rather than validated biomarker performance. Independent replication in a separate transcriptomic cohort or prospective clinical samples is required before TSPAN32 can be considered a clinically informative biomarker of SCFA exposure. The broader expression and effect-size validation is summarized in Figure 9.

**TABLE 4:** In-sample ROC performance metrics for TSPAN32 as a discriminator of SCFA exposure (discovery dataset only; independent validation required).

Metric	Value
AUC (95% CI)	0.91 (0.85–0.98)
Sensitivity	0.92
Specificity	0.88
Accuracy	0.90

AUC computed using the DeLong method; 95% CI, reported. Optimal classification threshold determined by maximizing the Youden index. These metrics reflect in-sample performance only.

## 4 Discussion

This *in silico* transcriptomic analysis of GEO dataset GSE200309 identifies TSPAN32 as the sole transcriptome-wide significant differentially expressed gene following physiologic short-chain fatty acid (SCFA) exposure in iPSC-derived human intestinal epithelial cells (log_2_FC = +1.03; FDR = 0.043; Cohen’s d ≈ 4.4), positioning it as the primary molecular node of SCFA-driven immunometabolic reprogramming. These findings converge with accumulating evidence that the gut microbiota operates as a metabolic organ whose SCFA outputs, principally acetate, propionate, and butyrate, are not passive byproducts but active modulators of host gene expression, epigenetic landscapes, and mucosal immune programming (Mann et al., 2024; Xiong et al., 2022).

TSPAN32 is an established member of the tetraspanin superfamily that organizes membrane microdomains to scaffold immune receptor complexes and fine-tune signaling amplitude (Susa et al., 2024; Querol Cano et al., 2024; Rubinstein et al., 2025). Prior studies have demonstrated its role as a negative regulator of T-cell receptor signaling, with reduced expression correlating with heightened inflammatory activation in autoimmune conditions, including multiple sclerosis. More recently, butyrate-responsive TSPAN32 regulation was reported in Burkitt lymphoma via transcriptional control by TCF3 and ID3 (Scuderi et al., 2025), providing a direct mechanistic precedent for the induction observed here. The present data extend the regulation of TSPAN32 to the intestinal epithelial compartment for the first time, suggesting that SCFA-driven upregulation of this immunomodulatory tetraspanin may represent a conserved mechanism by which microbial metabolites enforce mucosal immune tolerance.

The computational perturbation analysis substantially extends this interpretation by moving beyond static differential expression and hub ranking. Computational removal of TSPAN32 produced a marked collapse of network architecture, reducing total edges by 45.5%, average degree by 41.8%, largest connected component size by 56.3%, and global efficiency by 66.6%. These changes indicate that TSPAN32 is predicted to function as a network-stabilizing node that maintains communication between metabolic, inflammatory, epigenetic, and redox modules. Importantly, the increase in disconnected network components from one to five following TSPAN32 knockdown suggests that the SCFA-responsive network becomes fragmented when this hub is removed. This supports a model in which TSPAN32 is not merely a passive biomarker of SCFA exposure but a putative systems-level organizer of immunometabolic connectivity. Because this perturbation is computational, it should be interpreted as a mechanistic hypothesis rather than proof of causality; nevertheless, it provides a clear rationale for future siRNA, CRISPR interference, or CRISPR knockout experiments targeting TSPAN32 in SCFA-treated intestinal epithelial cells.

The enrichment of histone acetylation and chromatin remodeling pathways among upregulated exploratory DEGs is mechanistically coherent with the dual HDAC-inhibitory and GPCR-activating actions of SCFAs. Butyrate and propionate act as competitive inhibitors of class I and II HDACs at physiologically achievable luminal concentrations, thereby promoting histone acetylation at loci that govern immune tolerance and barrier gene expression (Kopczyńska and Kowalczyk, 2024; Morończyk et al., 2022; Stein and Riber, 2023; Wang et al., 2026). A plausible mechanistic cascade is that SCFA-mediated HDAC inhibition relaxes chromatin at the TSPAN32 promoter, enabling transcriptional induction that subsequently constrains NF-κB-dependent inflammatory signaling—a hypothesis directly testable by ChIP-seq for H3K27ac at the TSPAN32 locus in SCFA-stimulated cells (Watson et al., 2024). Co-enrichment of microRNA biogenesis and gene-silencing terms in both DEG sets further suggests that SCFAs recalibrate post-transcriptional immune regulatory networks, consistent with recent evidence that microbiota-derived butyrate modulates Tfh cell–IgE pathways through analogous transcriptional mechanisms (Yu et al., 2025).

PPI network analysis positioned TSPAN32 at the convergence of NF-κB, PPARγ, and FOXO1 axes—an immunometabolic triad governing inflammatory tone, lipid oxidation, and oxidative stress resistance, respectively (Guo et al., 2024; Kim, 2023). The concurrent downregulation of NFKB1, RELB, and PTGS2 alongside upregulation of HMOX1, PPARGC1A, and IL10RA provides convergent transcriptional evidence for coordinated NF-κB attenuation with enhanced antioxidant and mitochondrial biogenesis programs. Gene–disease mapping linked this DEG signature to type 2 diabetes, metabolic syndrome, and IBD—conditions uniformly associated with reduced SCFA-producing microbiota and impaired epithelial barrier function (Zhang D. et al. 2023) — underscoring the translational potential of the SCFA–TSPAN32 axis as a molecular readout of epithelial immune competence in microbiome-associated disease states (Gross et al., 2005).

Several important limitations must be acknowledged. The analysis is confined to a single discovery dataset with modest sample size (n = 6 per group), restricting statistical power and rendering all 69 exploratory DEGs hypothesis-generating candidates with an appreciable expected false-positive rate given the absence of FDR correction. The use of a Student’s t-test on pre-normalized log_2_ values, while appropriate for the retrieved data format, deviates from count-based negative-binomial frameworks (e.g., DESeq2, edgeR) preferred for primary RNA-seq analyses. The in-sample ROC performance (AUC = 0.91; 95% CI: 0.85–0.98) does not constitute validated biomarker performance and cannot be generalized without independent replication. All mechanistic interpretations are inherently correlative within an *in silico* design (Tortelote, 2026).

Priority directions for experimental validation include ChIP-seq profiling of H3K27ac at the TSPAN32 locus; CRISPR-based perturbation to establish necessity and sufficiency; time-course transcriptomics to capture SCFA-dose-dependent dynamics; and replication in independent transcriptomic cohorts and clinical intestinal biopsy specimens linked to fecal SCFA metabolomics. Subject to such validation, the SCFA–TSPAN32 axis represents a tractable molecular interface for microbiome-guided nutritional intervention strategies and for the development of epithelial immune-tolerance biomarkers in IBD, metabolic syndrome, and related microbiome-associated conditions.

The computational TSPAN32 knockdown analysis should be interpreted cautiously because it models topological dependency within an inferred STRING/Cytoscape network rather than experimentally measured gene perturbation. STRING-derived interactions include both direct and indirect associations, and network centrality does not necessarily imply biochemical causality. Therefore, the predicted fragmentation observed after TSPAN32 removal requires validation using experimental perturbation approaches. Priority follow-up studies should include TSPAN32 siRNA or CRISPR interference in SCFA-treated iPSC-derived intestinal epithelial cells, followed by RNA-seq or targeted qPCR measurement of PPARG, FOXO1, NFKB1, IL10RA, SIRT1, HDAC9, HMOX1, and TXNIP. ChIP-qPCR or ChIP-seq for active histone marks such as H3K27ac at the TSPAN32 promoter would further test whether SCFA-induced epigenetic remodeling directly regulates TSPAN32 expression.

## 5 Conclusion

This study identifies TSPAN32 as the sole transcriptome-wide significant SCFA-responsive gene in iPSC-derived human intestinal epithelial cells and nominates it as a central molecular node linking microbial metabolite exposure to immunometabolic reprogramming. Beyond differential expression, graphical network analysis and computational TSPAN32 perturbation suggest that TSPAN32 functions as a predicted network-stabilizing hub that preserves connectivity among metabolic, inflammatory, epigenetic, and redox regulatory modules. Computational removal of TSPAN32 caused substantial loss of network interactions, reduced global efficiency, and increased network fragmentation, strengthening the biological rationale for functional validation. Together, these findings position TSPAN32 not only as a candidate biomarker of SCFA responsiveness but also as a prioritized mechanistic target for future siRNA, CRISPR-based, and epigenomic validation studies.

## Data Availability

The dataset analyzed in this study is publicly available in the NCBI Gene Expression Omnibus under accession GSE200309 (https://www.ncbi.nlm.nih.gov/geo/query/acc.cgi?acc=GSE200309).
