# Supplementary material for: In silico transcriptomic analysis nominates TSPAN32 as a central node of SCFA-driven immunometabolic reprogramming in intestinal epithelial cells
Source: Front Bioinform. 2026 Sep 8;6:1847552. doi: 10.3389/fbinf.2026.1847552 (PMC13593874; doi:10.3389/fbinf.2026.1847552)
Supplement: Supplementary file 1 [file Supplementaryfile1.docx]

**SUPPLEMENTARY TABLES S1 AND S2**

**Supplementary Table S1.** Complete archive-derived list of 69 exploratory differentially expressed records following SCFA treatment

| **Original gene ID** | **Verified symbol** | **log₂FC** | **Adjusted p (FDR)** | **Cohen’s d** | **Direction** | **Status** |
| --- | --- | --- | --- | --- | --- | --- |
| Gene_0875 | TSPAN32 | +1.029 | 0.002 | +4.403 | Up | FDR-significant |
| Gene_0341 | — | -1.479 | 0.0868 | -4.143 | Down | Exploratory |
| Gene_0928 | — | +0.621 | 0.2874 | +2.855 | Up | Exploratory |
| Gene_0887 | — | +0.547 | 0.2874 | +2.881 | Up | Exploratory |
| Gene_0506 | — | +0.643 | 0.2874 | +2.887 | Up | Exploratory |
| Gene_0973 | — | +1.263 | 0.5239 | +2.680 | Up | Exploratory |
| Gene_0236 | — | -1.206 | 0.5239 | -2.879 | Down | Exploratory |
| Gene_0241 | — | -0.752 | 0.5794 | -2.276 | Down | Exploratory |
| Gene_0629 | — | +1.420 | 0.5794 | +2.755 | Up | Exploratory |
| Gene_0188 | — | -0.611 | 0.6545 | -2.280 | Down | Exploratory |
| Gene_0929 | — | +0.998 | 0.6677 | +2.313 | Up | Exploratory |
| Gene_0077 | — | -1.030 | 0.6677 | -2.305 | Down | Exploratory |
| Gene_0485 | — | -1.076 | 0.6677 | -2.106 | Down | Exploratory |
| Gene_0880 | — | -1.017 | 0.6677 | -2.144 | Down | Exploratory |
| Gene_0218 | — | -0.839 | 0.6677 | -2.253 | Down | Exploratory |
| Gene_0812 | — | +0.862 | 0.6677 | +1.959 | Up | Exploratory |
| Gene_0535 | — | -1.230 | 0.6677 | -1.959 | Down | Exploratory |
| Gene_0815 | — | +0.440 | 0.6677 | +2.012 | Up | Exploratory |
| Gene_0700 | — | +0.487 | 0.6677 | +1.995 | Up | Exploratory |
| Gene_0162 | — | +1.456 | 0.6677 | +2.189 | Up | Exploratory |
| Gene_0031 | — | +0.727 | 0.6677 | +1.926 | Up | Exploratory |
| Gene_0420 | — | -0.742 | 0.6677 | -1.854 | Down | Exploratory |
| Gene_0149 | — | -0.598 | 0.6677 | -1.841 | Down | Exploratory |
| Gene_0518 | — | +0.824 | 0.6677 | +1.944 | Up | Exploratory |
| Gene_0655 | — | -0.447 | 0.6745 | -1.888 | Down | Exploratory |
| Gene_0563 | — | -0.626 | 0.6819 | -1.792 | Down | Exploratory |
| Gene_0214 | — | +1.003 | 0.6819 | +1.850 | Up | Exploratory |
| Gene_0139 | — | -1.421 | 0.6819 | -1.914 | Down | Exploratory |
| Gene_0745 | — | -0.516 | 0.6819 | -1.891 | Down | Exploratory |
| Gene_0868 | — | +0.475 | 0.6819 | +1.752 | Up | Exploratory |
| Gene_0849 | — | +0.513 | 0.6819 | +1.676 | Up | Exploratory |
| Gene_0186 | — | -1.147 | 0.6819 | -1.845 | Down | Exploratory |
| Gene_0049 | — | -0.511 | 0.6819 | -1.672 | Down | Exploratory |
| Gene_0076 | — | -1.103 | 0.6819 | -1.698 | Down | Exploratory |
| Gene_0124 | — | -0.726 | 0.6819 | -1.659 | Down | Exploratory |
| Gene_0839 | — | -0.606 | 0.6819 | -1.666 | Down | Exploratory |
| Gene_0024 | — | -0.791 | 0.6819 | -1.628 | Down | Exploratory |
| Gene_0724 | — | +0.557 | 0.6819 | +1.633 | Up | Exploratory |
| Gene_0242 | — | -1.129 | 0.6819 | -1.626 | Down | Exploratory |
| Gene_0528 | — | +0.572 | 0.6819 | +1.663 | Up | Exploratory |
| Gene_0295 | — | +0.625 | 0.6819 | +1.696 | Up | Exploratory |
| Gene_0350 | — | -1.022 | 0.6819 | -1.611 | Down | Exploratory |
| Gene_0508 | — | +0.581 | 0.6819 | +1.692 | Up | Exploratory |
| Gene_0223 | — | +0.377 | 0.6819 | +1.571 | Up | Exploratory |
| Gene_0333 | — | +0.477 | 0.6819 | +1.654 | Up | Exploratory |
| Gene_0900 | — | -0.910 | 0.6819 | -1.632 | Down | Exploratory |
| Gene_0979 | — | -0.764 | 0.6819 | -1.522 | Down | Exploratory |
| Gene_0938 | — | +0.520 | 0.6819 | +1.510 | Up | Exploratory |
| Gene_0883 | — | -1.473 | 0.6819 | -1.722 | Down | Exploratory |
| Gene_0758 | — | -0.625 | 0.6819 | -1.504 | Down | Exploratory |
| Gene_0721 | — | -0.806 | 0.6819 | -1.505 | Down | Exploratory |
| Gene_0981 | — | +0.905 | 0.6819 | +1.600 | Up | Exploratory |
| Gene_0035 | — | -0.636 | 0.6819 | -1.612 | Down | Exploratory |
| Gene_0949 | — | -0.535 | 0.6819 | -1.482 | Down | Exploratory |
| Gene_0509 | — | -1.071 | 0.6819 | -1.548 | Down | Exploratory |
| Gene_0025 | — | +0.476 | 0.6819 | +1.508 | Up | Exploratory |
| Gene_0611 | — | -0.308 | 0.6819 | -1.492 | Down | Exploratory |
| Gene_0813 | — | -0.516 | 0.6819 | -1.478 | Down | Exploratory |
| Gene_0382 | — | +0.456 | 0.6819 | +1.503 | Up | Exploratory |
| Gene_0704 | — | -0.914 | 0.6819 | -1.482 | Down | Exploratory |
| Gene_0286 | — | -0.759 | 0.6819 | -1.602 | Down | Exploratory |
| Gene_0070 | — | -0.525 | 0.6819 | -1.469 | Down | Exploratory |
| Gene_0195 | — | -0.494 | 0.6819 | -1.498 | Down | Exploratory |
| Gene_0853 | — | +0.585 | 0.6819 | +1.599 | Up | Exploratory |
| Gene_0391 | — | +0.451 | 0.6819 | +1.440 | Up | Exploratory |
| Gene_0691 | — | -0.517 | 0.6819 | -1.500 | Down | Exploratory |
| Gene_0651 | — | +0.660 | 0.6819 | +1.540 | Up | Exploratory |
| Gene_0445 | — | +0.689 | 0.6819 | +1.502 | Up | Exploratory |
| Gene_0002 | — | +0.747 | 0.6819 | +1.448 | Up | Exploratory |

**Supplementary Table S2.** Manuscript-reported functional enrichment findings for exploratory DEGs

| **DEG set** | **Enrichr library/category** | **Reported enriched process or pathway** | **Reported significance** | **Manuscript interpretation** |
| --- | --- | --- | --- | --- |
| Upregulated (n = 31) | GO Biological Process | Positive regulation of microRNA biogenesis | Adjusted p < 1 × 10⁻⁶ | Post-transcriptional regulation |
| Upregulated (n = 31) | GO Biological Process | Chromatin remodeling | Adjusted p < 1 × 10⁻⁶ | SCFA-associated epigenetic regulation |
| Upregulated (n = 31) | GO Biological Process | Gene silencing via histone modification | Adjusted p < 1 × 10⁻⁶ | Histone-modification-related response |
| Downregulated (n = 38) | GO Biological Process | Negative regulation of extrinsic apoptotic signaling | Adjusted p < 5 × 10⁻³ | Attenuated apoptotic signaling |
| Downregulated (n = 38) | GO Biological Process | Response to organic cyclic compounds | Adjusted p < 5 × 10⁻³ | Response to organic cyclic compounds |
| Downregulated (n = 38) | Pathway category* | Attenuation of inflammatory response pathways | Adjusted p < 5 × 10⁻³ | Anti-inflammatory response |
